# Successful Conservative Management of a Dislocated IUD

**DOI:** 10.1155/2015/130528

**Published:** 2015-03-12

**Authors:** Hasan Ali Inal, Zeynep Ozturk Inal, Ender Alkan

**Affiliations:** ^1^Department of Gynaecology and Obstetrics, Konya Research and Training Hospital, Meram, 04220 Konya, Turkey; ^2^Department of Radiology, Konya Research and Training Hospital, Meram, 04220 Konya, Turkey

## Abstract

*Background*. Intrauterine contraceptive devices (IUDs) are widely utilized all over the world owing to their low cost and high efficacy. Uterine perforation is a rare complication that may occur at IUD insertion resulting in extrauterine location of the IUD. Traditionally, surgical removal of dislocated IUDs has been recommended. *Case*. A 68-year-old patient who had an IUD (Lippes loop) inserted 32 years ago and whose routine examination incidentally revealed a dislocated IUD in the abdominal cavity. The patient remained asymptomatic during three years of follow-up and the IUD was left in place. *Conclusion*. Asymptomatic patients, whose vaginal examinations and ultrasonography or X-ray results reveal a dislocated IUD, may benefit from conservative management.

## 1. Introduction

Intrauterine contraceptive devices (IUDs) are highly effective, safe, convenient, and the most popular reversible birth control method that are used by about 100 million women all over the world [1]. In Turkey, it has been estimated that 17% of women in the reproductive age use copper-releasing IUDs and levonorgestrel-releasing intrauterine systems that are inserted by certified gynecologists, midwives, and medical practitioners [2].

Problems associated with the use of IUD include infection, uterine bleeding, pelvic abscess, and uterine perforation. The uterine perforation rate has been reported to be 1.6 per 1,000 applications [3]. This complication usually occurs at the time of initial IUD insertion but the diagnosis is very often delayed.

The World Health Organization (WHO) suggested that all dislocated IUDs have to be removed promptly due to the risk of bowel perforation [4]. Recently it has also been suggested that the dislocated IUD should be surgically removed in symptomatic patients while conservative management is suggested for asymptomatic patients [5]. While, in the past, a dislocated IUD was surgically removed via laparotomy, today, a dislocated IUD can usually be safely removed using laparoscopy [6].

We here report a patient with a dislocated IUD that was found in the abdominal cavity in whom surgical intervention was not considered necessary.

## 2. The Case

**Figure 1:**
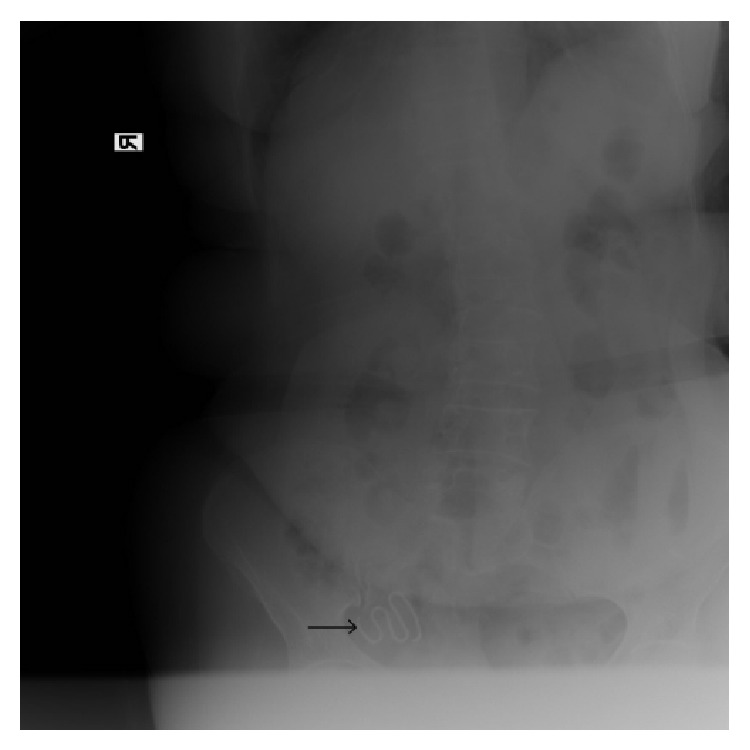
Pelvic X-ray reveals a Lippes loop IUD in the pelvis.

**Figure 2:**
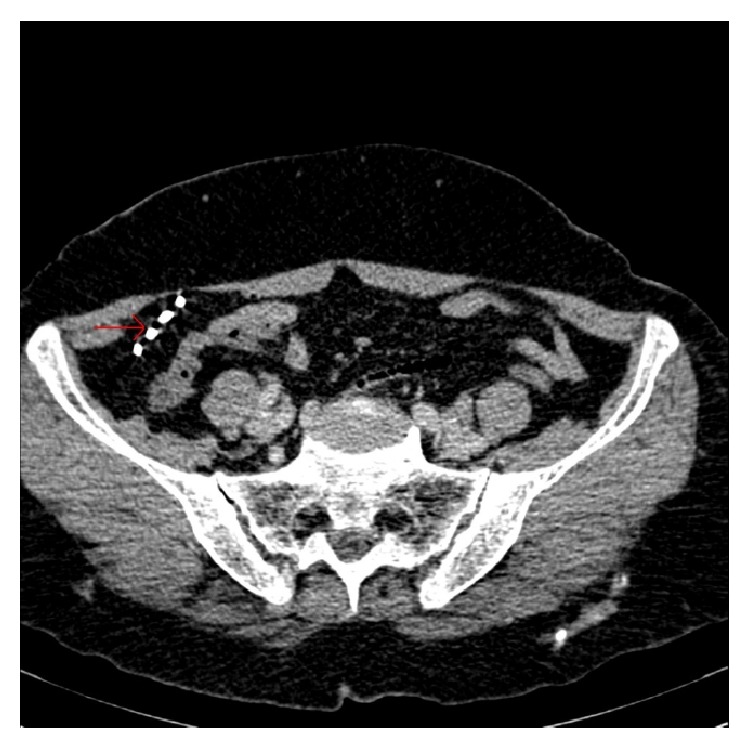
CT scan shows the intrauterine device in the right upper front part of the abdomen.

A 68-year-old woman, gravida 4 and para 3 with one spontaneous abortion and with no significant medical history, presented to the menopausal clinic for a routine check. She had a Lippes loop IUD inserted 32 years ago and she did not have it checked for many years. Her previous gynecological examination was performed 16 years ago. Standard vaginal and physical examinations revealed normal findings and all laboratory findings including a complete blood count and blood chemistry profile were within normal levels. The IUD strings were not visible. The IUD was not detected by abdominal and pelvic transvaginal ultrasonography. Therefore, we suspected that the IUD might have dislocated. Abdominopelvic X-ray in anteroposterior position and computed tomography (CT) scan results revealed the dislocated IUD in the right lower front part of the abdomen and no pathologies were found in the pelvic genital structures (Figures 1 and 2).

As the patient did not have any symptoms, we did not perform any surgical attempt (laparoscopy or laparotomy) to remove the dislocated IUD. We observed the patient for three years during which no signs nor symptoms related to the dislocated IUD were observed.

## 3. Discussion

The risk of uterine perforation related to IUD insertion varies between 0.60 and 0.87 per 1,000 insertions depending on the timing of the insertion, the skill of the performing physician, the position of the uterus (anteverted or retroverted), or the presence of a uterine anomaly [7]. Uterine perforation occurs most frequently at the time of insertion [4]. Studies have shown that more than 90% of uterine perforations occur when an IUD is inserted within the first postpartum year during the breast-feeding period [2, 8]. While a patient with a dislocated extrauterine IUD may be diagnosed at a health center to which she has presented with symptoms of lower abdominal pain, pregnancy, or irregular menstruation, IUD dislocation can also be incidentally diagnosed during routine checks without preceding symptoms or signs.

If dislocation of an IUD is suspected, vaginal examination and transvaginal ultrasound can usually reveal that the IUD is not located in the uterine cavity. In order to exactly locate the IUD, pelvic ultrasonography or abdominopelvic X-ray or, if those methods fail, advanced imaging methods (computerized tomography or magnetic resonance imaging) should be utilized [4, 7].

Phupong et al. [9] suggested that uterine perforations were brought about by uterine contractions as a result of infection and gave way to peritonitis and the authors consequently argued that a dislocated IUD could damage adjacent organs in the form of perforation of bowel and bladder, causing intestinal obstruction and pelvic abscess.

The WHO IUD report as well as some studies recommends that a dislocated IUD should be removed because of potential problems with bowel injury, chronic pelvic pain, and infertility [4, 6, 10]. The preferred method for the removal of a dislocated IUD is laparoscopy but laparotomy may have to be performed in some cases. Adoni and Chetrit reported no negative conditions such as adhesion formation when they evaluated 11 patients with dislocated IUDs (4 with Lippes loops, 7 with copper-bearing IUDs: 3 Multiload; 4 Nova-T) [11]. The authors argued that copper-IUDs or levonorgestrel-releasing IUSs caused less complications and it was not mandatory to remove them. Similarly, Markovitch et al. evaluated 3 patients with dislocated IUDs laparoscopically and reported no adhesion formations [5]. Studies have demonstrated that adhesions are formed right after the perforation around the dislocated IUD and limited to that area [9, 11]. It has also been argued that adhesion formation might be more generalized in the event of surgical procedures like laparoscopy or laparotomy [12].

When reviewing the literature, it became apparent that a dislocated IUD did not always need to be removed in an asymptomatic patient, despite the WHO recommendation. Since our patient had been asymptomatic for a very long time and, moreover, did not wish to undergo surgical intervention, her condition was regularly monitored for three years and no problems were observed.

## 4. Conclusion

A dislocated IUD in an asymptomatic patient does not need to be surgically removed. Such a patient might benefit from conservative management.
